# Crystal structure of 1-(2,6-diiso­propyl­phen­yl)-1*H*-imidazole

**DOI:** 10.1107/S2056989023009179

**Published:** 2023-10-26

**Authors:** Neil Dudeja, Briana C. Arreaga, Jacob P. Brannon, S. Chantal E. Stieber

**Affiliations:** a Department of Chemistry & Biochemistry, California State Polytechnic University, Pomona, 3801 W. Temple Ave., Pomona, CA 91768, USA; Harvard University, USA

**Keywords:** crystal structure, DippIm, imidazole, aryl imidazole

## Abstract

The crystal structure of the title compound, C_15_H_20_N_2_ or ^Dipp^Im, is reported. At 106 (2) K, the mol­ecule has monoclinic *P*2_1_/c symmetry with four mol­ecules in the unit cell. The imidazole ring is rotated 80.7 (1)° relative to the phenyl ring. Inter­molecular stabilization primarily results from close contacts between the N atom at the 3-position on the imidazole ring and the C—H bond at the 4-position on the neighboring ^Dipp^Im, with ar­yl–aryl distances outside of the accepted distance of 5 Å for π-stacking.

## Abstract 2

At 106 (2) K, the title mol­ecule has monoclinic *P*2_1_/c symmetry with four mol­ecules in the unit cell. The imidazole ring is rotated 80.7 (1)° relative to the phenyl ring. Inter­molecular stabilization primarily results from close contacts between the N-atom at the 3-position on the imidazole ring and the C—H bond at the 4-position on the neighboring ^Dipp^Im.

## 1. Chemical context

Imidazoles are stable aromatic heterocyclic compounds comprised of a five-membered heterocycle containing two non-adjacent nitro­gen atoms and three carbon atoms. They are precursors in many synthetic processes and find use in pharmaceuticals and agrochemicals to create anti­fungal agents and fungicides (Ebel *et al.*, 2000 ▸). 1-(2,6-Diiso­propyl­phen­yl)-1*H*-imidazole (^Dipp^Im) additionally has an aryl ring attached to the imidazole.

Several synthetic approaches towards the synthesis of ^Dipp^Im are reported, with the most common current route being through the one-pot synthesis with glyoxal, formaldehyde, ammonium chloride, and 2,6-diisopropyl aniline, followed by an acidic workup with H_3_PO_4_ (Liu *et al.*, 2003 ▸). A disadvantage of this general route is that the yields are often low, especially for more hindered imidazoles. An alternative approach followed an Ullmann-type coupling using 2-iodo-1,3-diiso­propyl­benzene and imidazole, with 10% CuI, 40% *N*,*N*′-di­methyl­ethylenedi­amine, and Cs_2_CO_3_, but only resulted in 19% yield of ^Dipp^Im (Alcalde *et al.*, 2005 ▸). The highest yield approach with 78% yield was originally reported in 1889 and is from the reaction of 2,6-diisopropyl aniline with thio­phosgene (Cl_2_CS) in H_2_O, followed by addition of H_2_NCH_2_CH(OEt_2_), and acidic workup with HCl and HNO_3_ (Wohl & Marckwald, 1889 ▸; Johnson *et al.*, 1969 ▸). Despite being the first reported method, this synthetic approach is significantly concerning from a chemical safety perspective because thio­phosgene is highly toxic.

^Dipp^Im is often used as a precursor to a variety of *N*-heterocyclic carbene (NHC) ligands, which are a common ligand class for organometallic chemistry and catalysis (Arduengo, 1999 ▸; Hopkinson *et al.*, 2014 ▸; Lumiss *et al.*, 2015 ▸). To create monodentate NHC ligands, an imidazole is typically reacted with an alkyl or aryl halide to form an imidazolium salt. For bidentate NHC ligands, two imidazoles can be reacted with an alkyl or aryl dihalide to form a bis­(imidazolium) salt (Gardiner *et al.*, 1999 ▸; Thompson *et al.*, 2022 ▸). These imidazolium salts are then deprotonated by a base such as sodium *tert*-butoxide (NaOtBu) or potassium bis­(tri­methyl­sil­yl)amide (KHMDS) to form the free carbene ligands (Brendel *et al.*, 2014 ▸; Yamamoto *et al.*, 2018 ▸).

Few aryl­imidazoles have been structurally characterized, with 1-(2,4,6-tri­methyl­phen­yl)-1*H*-imidazole (^Mes^Im) reported by our group (Brannon *et al.*, 2018 ▸). Herein, the crystallographic characterization of 1-(2,6-diiso­propyl­phen­yl)-1*H*-imidazole (^Dipp^Im) is reported. 
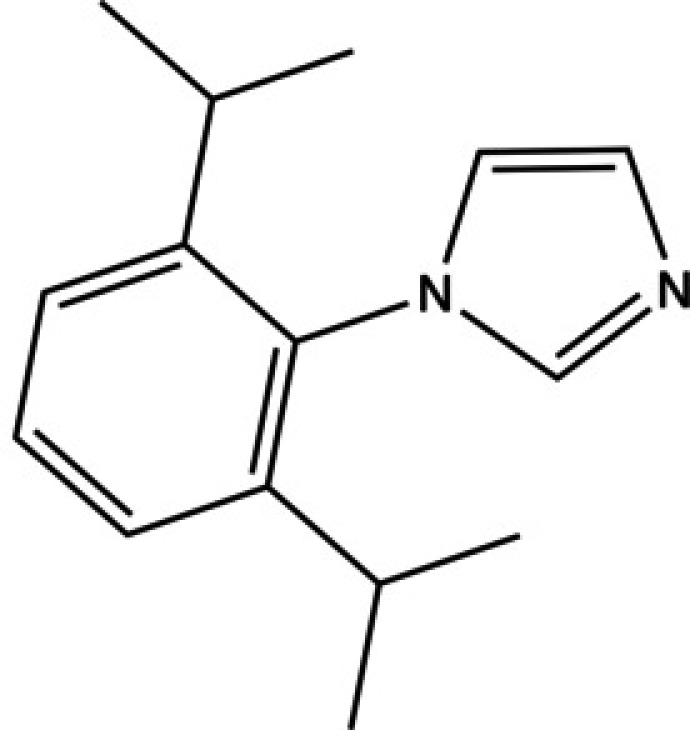


## 2. Structural commentary

**Figure 1:**
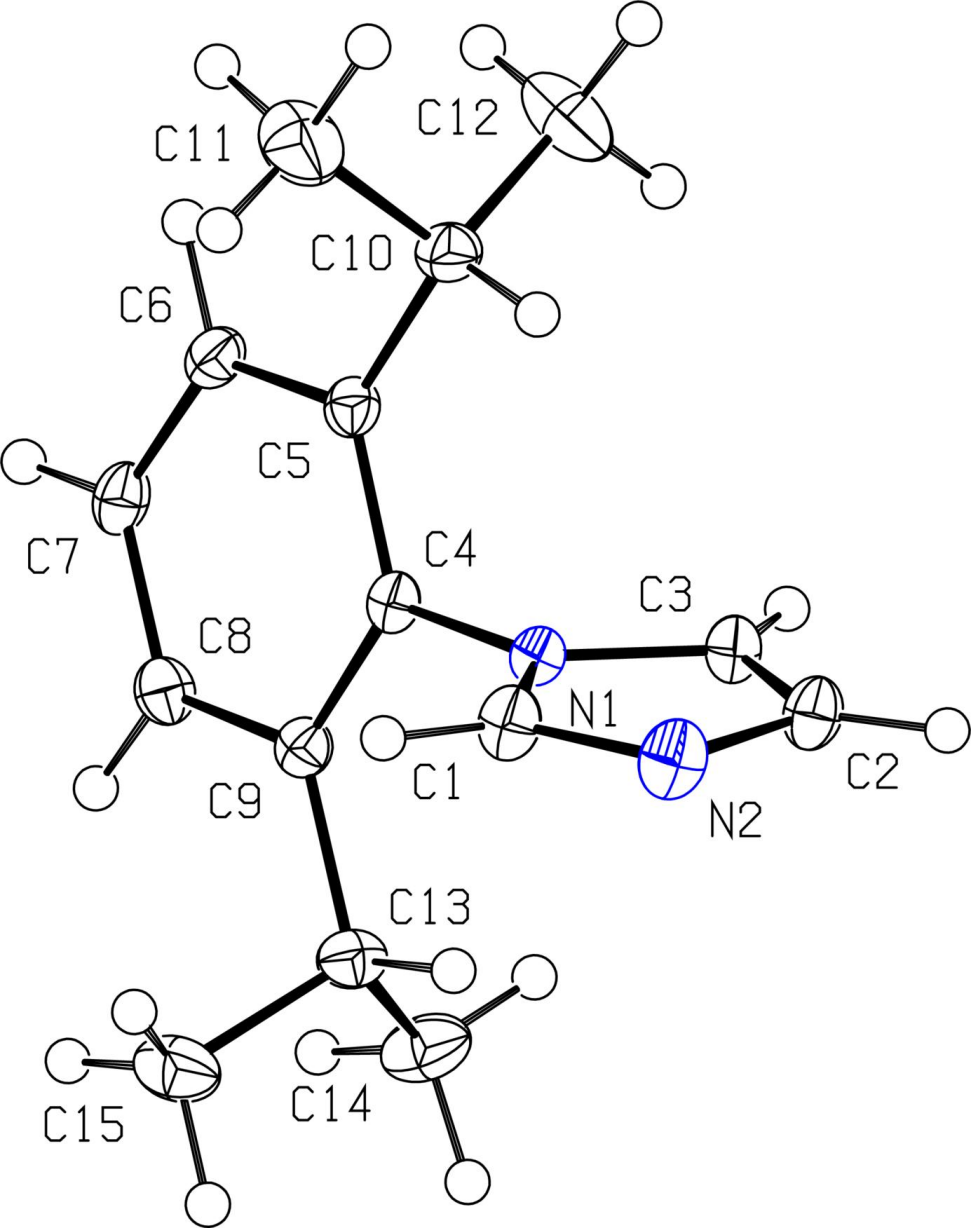
View of one mol­ecule of ^Dipp^Im with 50% probability ellipsoids.

^Dipp^Im crystallizes as depicted in Fig. 1 ▸ with a planar imidazole ring containing atoms N1, N2, and C1–C3. The bond angles within the five-membered imidazole ring are C1—N1—C3 = 107.02 (9)°, N1—C3—C2 = 105.30 (10)°, C3—C2—N2 = 110.95 (10)°, C2—N2—C1 = 104.73 (10)°, and N2—C1—N1 = 112.01 (10)°. These are all within error of the reported values for ^Mes^Im of 106.44 (16), 105.65 (17), 110.89 (18), 104.54 (17), and 112.48 (17)°, respectively (Brannon *et al.* 2018 ▸). These data suggest that changing the aryl group from 2,4,6-tri­methyl­phenyl to 2,6-diiso­propyl­phenyl has no significant effect on the imidazole ring.

Bond distances to C1 are consistent with a shorter bond of 1.3544 (14) Å between N1—C1 and a longer bond of 1.3153 (16) Å between C1—N2, likely due to steric effects of the aryl group. The backbone imidazole C2—C3 bond distance of 1.3578 (16) Å is consistent with a C*sp* ^2^=C*sp* ^2^ double bond in an imidazole ring (Allen *et al.*, 1987 ▸). The backbone N1—C3 and N2—C2 distances are consistent with C*sp* ^2^—N imidazole single bonds at 1.3769 (14) and 1.3759 (16) Å, respectively (Allen *et al.*, 1987 ▸). Comparable distances for ^Mes^Im are N1—C1 = 1.357 (3) Å, C1—N2 = 1.316 (3) Å, C2—C3 = 1.356 (3) Å, N1—C3 = 1.384 (2) Å, and N2—C2 = 1.382 (3) Å (Brannon *et al.*, 2018 ▸). The imidazole ring distances are comparable to those reported for ^Mes^Im, indicating that the bulkier aryl group has no significant effect.

## 3. Supra­molecular features

**Figure 2:**
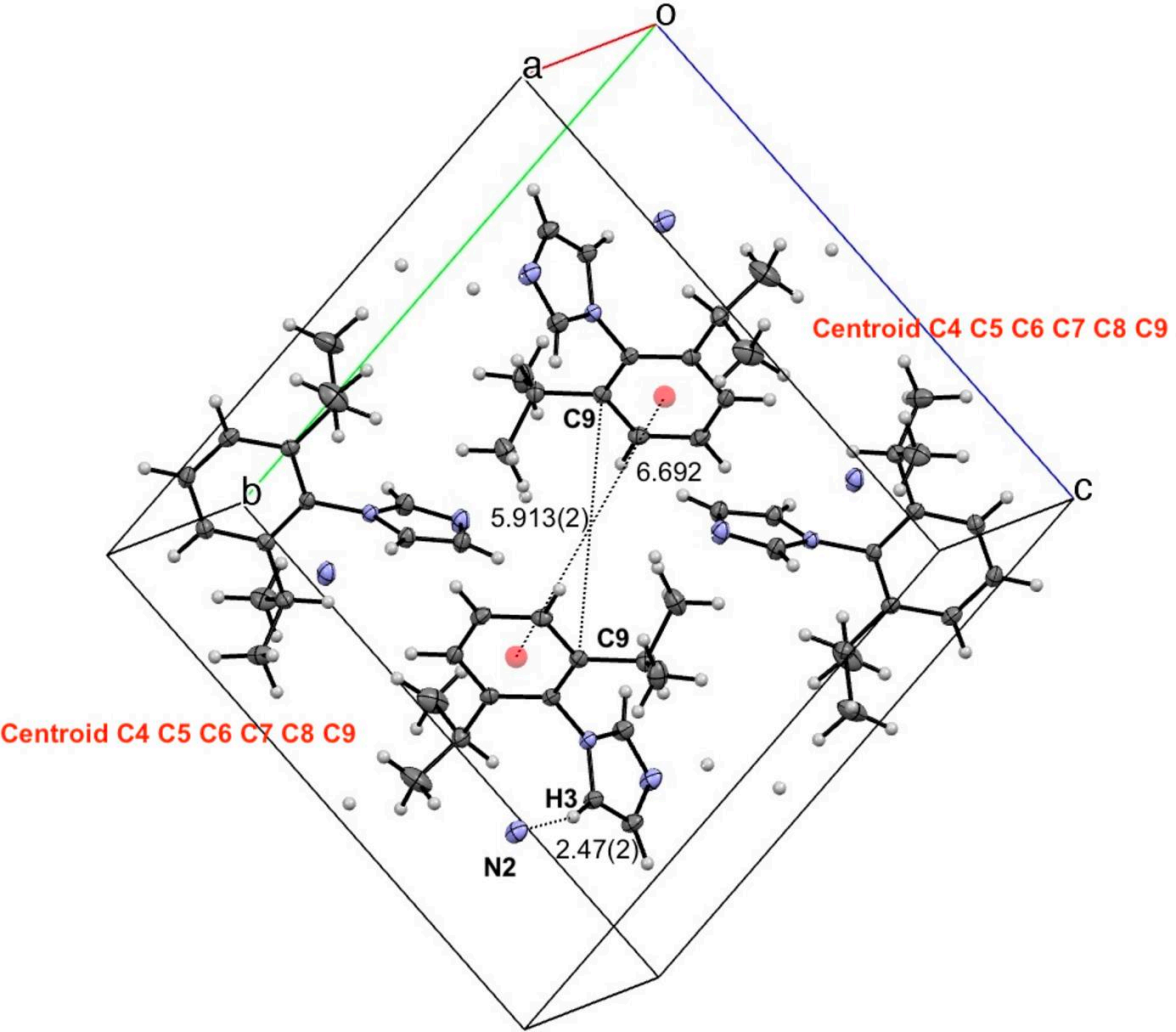
View of four mol­ecules of ^Dipp^Im in the unit cell with 50% probability ellipsoids, highlighting inter­molecular distances and close contacts. Distances between centroids (red circles) are listed without standard deviations because these positions were calculated.

The unit cell contains four full mol­ecules of 2,6-diiso­propyl­phenyl imidazole (Fig. 2 ▸). Each mol­ecule is oriented such that the imidazole groups are at 80.7 (1)° relative to the aryl ring, based on the measured C1—N1—C4—C9 torsion . Distances between aryl rings are 6.692 Å as measured between neighboring C4–C9 centroids, and 5.912 (2) Å as measured between C9–C9 on neighboring mol­ecules. There is no uncertainty in the distance between centroids, since these were placed using the *Mercury* program’s centroid algorithm (Macrae *et al.*, 2020 ▸). Both of these distances are greater than 5 Å, supporting no significant π-stacking stabilization (Janiak, 2000 ▸). The closest contact between neighboring mol­ecules is between N2⋯H3 at a distance of 2.47 (2) Å. This technically can be considered a hydrogen bond (Table 1 ▸) because H3 is bound to C3, which is bound to an electronegative atom, N1. Therefore, the supra­molecular structure of ^Dipp^Im is primarily stabilized through hydrogen bonding between neighboring imidazoles.

## 4. Database survey

A survey of the Cambridge Structural Database (Groom *et al.*, 2016 ▸) on August 30, 2023 yielded no structural results for ^Dipp^Im through both a drawn structure search and a search of the full name 1-(2,6-diiso­propyl­phen­yl)-1*H*-imidazole. A SciFinder search (SciFinder, 2018 ▸) resulted in a substance match with code 25364-47-0, however no structural data were reported.

## 5. Synthesis and crystallization

**Figure 3:**
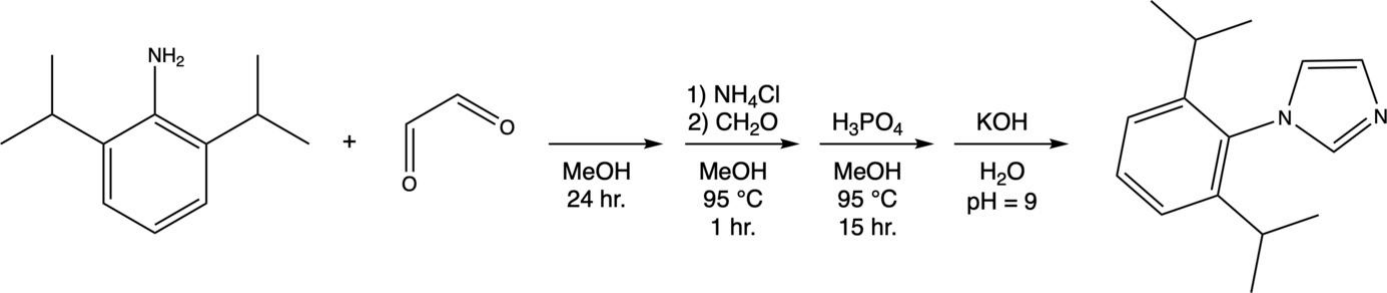
Reaction scheme.

The synthesis for ^Dipp^Im (Fig. 3 ▸) was adapted from a literature procedure (Liu *et al.*, 2003 ▸). A 500 mL three-necked round-bottomed flask was charged with 10.01 g (0.0564 mol, 1 eq.) of 2,6-diiso­propyl­aniline followed by 8.20 g (0.141 mol, 1 eq.) of 40% aqueous glyoxal and approximately 100 mL of methanol. The resulting color changed from a clear yellow to a rusty orange solution with a yellow precipitate. Using a funnel, 6.03 g (0.112 mol, 2 eq.) of ammonium chloride and 9.16 g (0.305 mol, 2 eq.) of 37% aqueous formaldehyde were added to the round-bottomed flask and diluted with 130 mL of methanol. The mixture was refluxed for 1 h at 368 K, resulting in a dark-brown solution. The flask was removed from the heat and cooled to room temperature before being placed in an ice bath to cool, followed by addition of 15 mL (0.15 mol, 2 eq.) of phospho­ric acid over the course of 12 minutes. After addition, it was refluxed at 368 K for 14.5 h, resulting in an opaque dark-red solution. The solution was cooled to room temperature and concentrated *in vacuo*. The dark-brown residue was poured over 300 g of ice and neutralized with a concentrated potassium hydroxide solution until the pH reached 9, resulting in a light-brown solution with a dark-brown precipitate. The mixture was extracted three times with approximately 100 mL of diethyl ether, washed 3 times with approximately 100 mL of water, and washed three times with approximately 100 mL of brine. The mixture was transferred to a 1 L round-bottom flask, dried with sodium sulfate, and left to dry for approximately 20 h, resulting in a dark-brown solution. The sodium sulfate was removed by gravity filtration and the solution was concentrated *in vacuo* resulting in a light-brown solid. The solid was then recrystallized with ethyl acetate, resulting in 1.33 g (10.4% yield) of colorless crystals. The product was characterized with ^1^H NMR and the results were consistent with reported literature values (Liu *et al.*, 2003 ▸).

## 6. Refinement

Crystal data, data collection and structure refinement details are summarized in Table 2 ▸. Hydrogen atoms were refined with all H-atom parameters.

## Supplementary Material

Crystal structure: contains datablock(s) I. DOI: 10.1107/S2056989023009179/oi2001sup1.cif

Structure factors: contains datablock(s) I. DOI: 10.1107/S2056989023009179/oi2001Isup2.hkl

Supporting information file. DOI: 10.1107/S2056989023009179/oi2001Isup3.cml

CCDC reference: 2302037

Additional supporting information:  crystallographic information; 3D view; checkCIF report

## Figures and Tables

**Table 1:** Hydrogen-bond geometry (Å, °)

*D*—H⋯*A*	*D*—H	H⋯*A*	*D*⋯*A*	*D*—H⋯*A*
C3—H3⋯N2^i^	0.966 (17)	2.474 (17)	3.416 (2)	164.9 (12)

Symmetry code: (i) 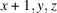 .

**Table 2:** Experimental details

Crystal data	
Chemical formula	C_15_H_20_N_2_
*M*_r_	228.33
Crystal system, space group	Monoclinic, *P*2_1_/*c*
Temperature (K)	106
*a*, *b*, *c* (Å)	5.6642 (13), 16.519 (6), 14.414 (6)
β (°)	90.73 (2)
*V* (Å^3^)	1348.6 (8)
*Z*	4
Radiation type	Mo *K*α
μ (mm^−1^)	0.07
Crystal size (mm)	0.20 × 0.15 × 0.10

Data collection	
Diffractometer	Bruker D8 Venture Kappa
Absorption correction	Multi-scan (*SADABS*; Krause *et al.*, 2015 ▸)
No. of measured, independent and observed [*I* > 2σ(*I*)] reflections	22918, 2975, 2750
*R*_int_	0.030
(sin θ/λ)_max_ (Å^−1^)	0.641

Refinement	
*R*[*F* ^2^ > 2σ(*F* ^2^)], *wR*(*F* ^2^), *S*	0.040, 0.099, 1.09
No. of reflections	2975
No. of parameters	234
H-atom treatment	All H-atom parameters refined
Δρ_max_, Δρ_min_ (e Å^−3^)	0.26, −0.23

Computer programs: *APEX3* and *SAINT* (Bruker, 2018 ▸), *SHELXT2014/5* (Sheldrick, 2015*a* ▸), *SHELXL2019/3/1* (Sheldrick, 2015*b* ▸), *OLEX2* (Dolomanov *et al.*, 2009 ▸), and *Mercury* (Macrae *et al.*, 2020 ▸).

## supplementary crystallographic information

### Crystal data

**Table:**

C_15_H_20_N_2_	*F*(000) = 496
*M**_r_* = 228.33	*D*_x_ = 1.125 Mg m^−^^3^
Monoclinic, *P*2_1_/*c*	Mo *K*α radiation, λ = 0.71073 Å
*a* = 5.6642 (13) Å	Cell parameters from 9863 reflections
*b* = 16.519 (6) Å	θ = 2.8–47.3°
*c* = 14.414 (6) Å	µ = 0.07 mm^−^^1^
β = 90.73 (2)°	*T* = 106 K
*V* = 1348.6 (8) Å^3^	Prism, colorless
*Z* = 4	0.20 × 0.15 × 0.10 mm

### Data collection

**Table:**

Bruker D8 Venture Kappa diffractometer	2750 reflections with *I* > 2σ(*I*)
Radiation source: microfocus sealed tube	*R*_int_ = 0.030
φ and ω scans	θ_max_ = 27.1°, θ_min_ = 2.8°
Absorption correction: multi-scan (SADABS; Krause *et al.*, 2015)	*h* = −7→7
	*k* = −21→21
22918 measured reflections	*l* = −18→18
2975 independent reflections	

### Refinement

**Table:**

Refinement on *F*^2^	0 restraints
Least-squares matrix: full	Hydrogen site location: difference Fourier map
*R*[*F*^2^ > 2σ(*F*^2^)] = 0.040	All H-atom parameters refined
*wR*(*F*^2^) = 0.099	*w* = 1/[σ^2^(*F*_o_^2^) + (0.0376*P*)^2^ + 0.5846*P*] where *P* = (*F*_o_^2^ + 2*F*_c_^2^)/3
*S* = 1.09	(Δ/σ)_max_ < 0.001
2975 reflections	Δρ_max_ = 0.26 e Å^−^^3^
234 parameters	Δρ_min_ = −0.23 e Å^−^^3^

### Special details

**Table:**

Geometry. All esds (except the esd in the dihedral angle between two l.s. planes) are estimated using the full covariance matrix. The cell esds are taken into account individually in the estimation of esds in distances, angles and torsion angles; correlations between esds in cell parameters are only used when they are defined by crystal symmetry. An approximate (isotropic) treatment of cell esds is used for estimating esds involving l.s. planes.

### Fractional atomic coordinates and isotropic or equivalent isotropic displacement parameters (Å^2^)

**Table:**

	*x*	*y*	*z*	*U*_iso_*/*U*_eq_	
N1	0.44635 (15)	0.73907 (5)	0.71450 (6)	0.0157 (2)	
H1	0.113 (3)	0.7150 (9)	0.6734 (10)	0.023 (3)*	
C1	0.2081 (2)	0.73808 (7)	0.72124 (8)	0.0215 (2)	
N2	0.13631 (17)	0.77200 (6)	0.79847 (7)	0.0245 (2)	
H2	0.337 (3)	0.8212 (9)	0.9035 (11)	0.028 (4)*	
C2	0.3404 (2)	0.79580 (7)	0.84352 (8)	0.0218 (2)	
C3	0.5334 (2)	0.77620 (7)	0.79331 (7)	0.0189 (2)	
H3	0.701 (3)	0.7824 (9)	0.8042 (10)	0.024 (3)*	
C4	0.58676 (18)	0.70733 (6)	0.64044 (7)	0.0155 (2)	
H4	0.518 (3)	0.8608 (9)	0.6295 (10)	0.028 (4)*	
C5	0.67446 (19)	0.76113 (6)	0.57433 (7)	0.0173 (2)	
H5	0.881 (3)	0.7657 (9)	0.4575 (10)	0.026 (4)*	
C7	0.8606 (2)	0.64725 (7)	0.49976 (7)	0.0204 (2)	
H7	0.806 (3)	0.5371 (9)	0.5612 (10)	0.028 (4)*	
C8	0.77076 (19)	0.59529 (7)	0.56604 (8)	0.0200 (2)	
H8	0.455 (3)	0.5972 (9)	0.7572 (10)	0.026 (4)*	
C9	0.63395 (18)	0.62423 (6)	0.63861 (7)	0.0171 (2)	
H9	0.928 (4)	0.8858 (12)	0.6469 (14)	0.059 (6)*	
H12	0.429 (3)	0.9356 (11)	0.4939 (12)	0.043 (4)*	
C12	0.8378 (3)	0.90170 (9)	0.59054 (13)	0.0409 (4)	
H11	0.794 (3)	0.9602 (11)	0.5977 (12)	0.045 (5)*	
C11	0.4803 (3)	0.87601 (8)	0.48960 (11)	0.0388 (3)	
C10	0.6171 (2)	0.85084 (7)	0.57667 (8)	0.0222 (2)	
H10	0.933 (3)	0.8961 (11)	0.5377 (13)	0.042 (5)*	
C15	0.3703 (2)	0.50568 (8)	0.66749 (10)	0.0305 (3)	
H16	0.457 (3)	0.4716 (11)	0.6197 (13)	0.049 (5)*	
H17	0.311 (3)	0.4685 (10)	0.7143 (11)	0.037 (4)*	
H18	0.829 (3)	0.4848 (10)	0.7182 (12)	0.042 (4)*	
H19	0.680 (3)	0.4898 (10)	0.8134 (12)	0.040 (4)*	
H20	0.856 (3)	0.5610 (10)	0.7892 (11)	0.036 (4)*	
H15	0.237 (3)	0.5350 (10)	0.6368 (11)	0.040 (4)*	
C14	0.7432 (2)	0.52320 (9)	0.76253 (10)	0.0314 (3)	
H14	0.337 (4)	0.8422 (13)	0.4783 (14)	0.061 (6)*	
C13	0.5411 (2)	0.56655 (7)	0.71156 (8)	0.0209 (2)	
H13	0.588 (3)	0.8716 (11)	0.4351 (14)	0.051 (5)*	
C6	0.81454 (19)	0.72946 (7)	0.50430 (7)	0.0196 (2)	
H6	0.954 (2)	0.6261 (8)	0.4495 (9)	0.020 (3)*	

### Atomic displacement parameters (Å^2^)

**Table:**

	*U*^11^	*U*^22^	*U*^33^	*U*^12^	*U*^13^	*U*^23^
N1	0.0140 (4)	0.0180 (4)	0.0150 (4)	0.0011 (3)	0.0003 (3)	−0.0003 (3)
C1	0.0145 (5)	0.0295 (6)	0.0205 (5)	0.0011 (4)	−0.0007 (4)	−0.0026 (4)
N2	0.0167 (5)	0.0336 (6)	0.0232 (5)	0.0032 (4)	0.0024 (4)	−0.0032 (4)
C2	0.0204 (5)	0.0268 (6)	0.0181 (5)	0.0024 (4)	0.0020 (4)	−0.0036 (4)
C3	0.0173 (5)	0.0224 (5)	0.0170 (5)	0.0004 (4)	−0.0004 (4)	−0.0029 (4)
C4	0.0127 (5)	0.0195 (5)	0.0144 (5)	0.0011 (4)	−0.0003 (4)	−0.0015 (4)
C5	0.0171 (5)	0.0191 (5)	0.0158 (5)	−0.0003 (4)	−0.0022 (4)	0.0000 (4)
C7	0.0181 (5)	0.0272 (6)	0.0162 (5)	0.0017 (4)	0.0027 (4)	−0.0041 (4)
C8	0.0196 (5)	0.0191 (5)	0.0214 (5)	0.0023 (4)	0.0004 (4)	−0.0030 (4)
C9	0.0151 (5)	0.0193 (5)	0.0169 (5)	−0.0001 (4)	−0.0009 (4)	0.0001 (4)
C12	0.0394 (8)	0.0226 (6)	0.0603 (10)	−0.0047 (6)	−0.0099 (7)	−0.0053 (6)
C11	0.0495 (9)	0.0227 (6)	0.0437 (8)	0.0063 (6)	−0.0175 (7)	0.0030 (6)
C10	0.0284 (6)	0.0177 (5)	0.0205 (5)	0.0011 (4)	0.0023 (4)	0.0019 (4)
C15	0.0263 (6)	0.0265 (6)	0.0387 (7)	−0.0063 (5)	−0.0001 (5)	0.0054 (5)
C14	0.0278 (6)	0.0359 (7)	0.0303 (6)	0.0002 (5)	−0.0021 (5)	0.0130 (6)
C13	0.0212 (5)	0.0194 (5)	0.0222 (5)	0.0012 (4)	0.0040 (4)	0.0026 (4)
C6	0.0185 (5)	0.0252 (6)	0.0151 (5)	−0.0027 (4)	0.0009 (4)	0.0015 (4)

### Geometric parameters (Å, º)

**Table:**

N1—C1	1.3544 (14)	C12—H9	0.99 (2)
N1—C3	1.3769 (14)	C12—H11	1.004 (18)
N1—C4	1.4382 (14)	C12—C10	1.5173 (19)
C1—H1	0.950 (15)	C12—H10	0.945 (18)
C1—N2	1.3153 (16)	C11—H12	1.028 (18)
N2—C2	1.3759 (16)	C11—C10	1.5245 (18)
C2—H2	0.961 (15)	C11—H14	1.00 (2)
C2—C3	1.3578 (16)	C11—H13	1.00 (2)
C3—H3	0.965 (15)	C10—H4	0.967 (15)
C4—C5	1.3988 (15)	C15—H16	1.019 (19)
C4—C9	1.3988 (15)	C15—H17	0.975 (17)
C5—C10	1.5176 (16)	C15—H15	0.998 (18)
C5—C6	1.3937 (16)	C15—C13	1.5280 (17)
C7—C8	1.3860 (16)	C14—H18	1.027 (18)
C7—C6	1.3845 (17)	C14—H19	0.988 (17)
C7—H6	0.969 (14)	C14—H20	0.968 (17)
C8—H7	0.984 (15)	C14—C13	1.5300 (17)
C8—C9	1.3941 (15)	C13—H8	0.968 (15)
C9—C13	1.5180 (15)	C6—H5	0.981 (15)
			
C1—N1—C3	107.02 (9)	H12—C11—H14	108.4 (15)
C1—N1—C4	127.58 (9)	H12—C11—H13	107.0 (14)
C3—N1—C4	125.40 (9)	C10—C11—H12	110.7 (10)
N1—C1—H1	120.6 (9)	C10—C11—H14	112.7 (12)
N2—C1—N1	112.01 (10)	C10—C11—H13	108.5 (11)
N2—C1—H1	127.4 (9)	H14—C11—H13	109.4 (16)
C1—N2—C2	104.73 (10)	C5—C10—H4	108.1 (9)
N2—C2—H2	121.7 (9)	C5—C10—C11	110.78 (10)
C3—C2—N2	110.95 (10)	C12—C10—H4	106.8 (9)
C3—C2—H2	127.4 (9)	C12—C10—C5	111.56 (11)
N1—C3—H3	121.5 (8)	C12—C10—C11	111.51 (12)
C2—C3—N1	105.30 (10)	C11—C10—H4	107.8 (9)
C2—C3—H3	133.2 (8)	H16—C15—H17	106.9 (14)
C5—C4—N1	118.59 (9)	H16—C15—H15	109.6 (14)
C9—C4—N1	118.64 (9)	H17—C15—H15	110.4 (14)
C9—C4—C5	122.75 (10)	C13—C15—H16	109.8 (11)
C4—C5—C10	121.82 (10)	C13—C15—H17	110.3 (10)
C6—C5—C4	117.73 (10)	C13—C15—H15	109.8 (10)
C6—C5—C10	120.44 (10)	H18—C14—H19	107.2 (13)
C8—C7—H6	120.1 (8)	H18—C14—H20	109.4 (13)
C6—C7—C8	120.25 (10)	H19—C14—H20	107.9 (13)
C6—C7—H6	119.6 (8)	C13—C14—H18	110.3 (9)
C7—C8—H7	118.6 (9)	C13—C14—H19	110.0 (10)
C7—C8—C9	121.15 (10)	C13—C14—H20	111.9 (10)
C9—C8—H7	120.3 (9)	C9—C13—H8	109.0 (8)
C4—C9—C13	122.34 (10)	C9—C13—C15	110.37 (10)
C8—C9—C4	117.31 (10)	C9—C13—C14	111.28 (10)
C8—C9—C13	120.34 (10)	C15—C13—H8	107.8 (9)
H9—C12—H11	107.3 (15)	C15—C13—C14	110.94 (11)
H9—C12—H10	109.9 (16)	C14—C13—H8	107.4 (9)
H11—C12—H10	108.8 (15)	C5—C6—H5	119.8 (8)
C10—C12—H9	112.2 (12)	C7—C6—C5	120.78 (10)
C10—C12—H11	109.9 (10)	C7—C6—H5	119.4 (8)
C10—C12—H10	108.6 (11)		
			
N1—C1—N2—C2	−0.11 (13)	C4—C5—C6—C7	1.25 (16)
N1—C4—C5—C10	−2.52 (15)	C4—C9—C13—C15	116.01 (12)
N1—C4—C5—C6	178.59 (9)	C4—C9—C13—C14	−120.37 (12)
N1—C4—C9—C8	−179.92 (9)	C5—C4—C9—C8	−1.27 (15)
N1—C4—C9—C13	0.36 (15)	C5—C4—C9—C13	179.01 (10)
C1—N1—C3—C2	−0.09 (12)	C7—C8—C9—C4	1.46 (16)
C1—N1—C4—C5	100.55 (13)	C7—C8—C9—C13	−178.81 (10)
C1—N1—C4—C9	−80.74 (14)	C8—C7—C6—C5	−1.08 (17)
C1—N2—C2—C3	0.05 (14)	C8—C9—C13—C15	−63.71 (14)
N2—C2—C3—N1	0.03 (13)	C8—C9—C13—C14	59.92 (14)
C3—N1—C1—N2	0.13 (13)	C9—C4—C5—C10	178.83 (10)
C3—N1—C4—C5	−79.97 (13)	C9—C4—C5—C6	−0.06 (16)
C3—N1—C4—C9	98.74 (13)	C10—C5—C6—C7	−177.65 (10)
C4—N1—C1—N2	179.68 (10)	C6—C5—C10—C12	−63.43 (15)
C4—N1—C3—C2	−179.66 (10)	C6—C5—C10—C11	61.42 (15)
C4—C5—C10—C12	117.71 (13)	C6—C7—C8—C9	−0.33 (17)
C4—C5—C10—C11	−117.43 (13)		

### Hydrogen-bond geometry (Å, º)

**Table:**

*D*—H···*A*	*D*—H	H···*A*	*D*···*A*	*D*—H···*A*
C3—H3···N2^i^	0.966 (17)	2.474 (17)	3.416 (2)	164.9 (12)

Symmetry code: (i) *x*+1, *y*, *z*.
